# The Chicago Charitable Eye and Ear Infirmary

**Published:** 1859-12

**Authors:** 


### THE CHICAGO CHARITABLE EYE AND EAR INFIRMARY.

During the six months, ending Nov. 1, 1859, *one hundred and eleven* patients have been treated by the surgeons of this Institution, making an aggregate of *two hundred and twenty-six* patients, who have been under their care since its organization.

This result shows a continued and increasing confidence in the Infirmary, on the part of those who seek treatment for diseases of the eye or ear, and must encourage its friends to continue their efforts to provide relief for this class of sufferers.

A charitable institution of this kind merits the interest and support of the public, since everywhere around us there are large numbers of poor afflicted with serious diseases of the eye, who are unable to meet the expense of suitable medical aid. Such patients are for a long time able to attend more or less to their usual labors, and consequently scarcely excite the notice or sympathy of the casual observer. In this condition, however, night and exposure frequently tend so much to increase their maladies, that they are soon unable to labor: the diseases are too often allowed to progress beyond the reach of treatment, and the sufferers, being incapable of gaining a support for themselves and families, are for ever after dependent upon their friends or the public.

To avert these evils, as far as possible, infirmaries have been established in nearly all the large cities of the civilized world, for the especial treatment of the poor, afflicted with ophthalmic diseases, and in many instances have been most richly endowed by public subscriptions.

From the fact, moreover, that eye infirmaries offer the means of preserving many sufferers from blindness, and consequently from being a burden to the State, as pupils in the Blind Asylum, the legislatures of several States have granted large sums of money for the support of these infirmaries, not only on the ground of humanity, but also of economy.

There is especial need of a good infirmary at Chicago. The city, already large, is rapidly increasing in weath and influence. The population, of which it is the commercial centre, is numbered by millions; few are aware how extensively severe affections of the eye prevail throughout the North-west. There is no public infirmary for these diseases in the Western States, and many patients, who can ill afford the expense, feel obliged to visit the Eastern cities for treatment.

It is hoped the physicians and the people of the State, will give the subject the serious thought the subject deserves.

*Officers of the Association.*—Walter L. Newberry, President; Charles V. Dyer and Luther Haven, Vice-Presidents; Samuel Stone, Secretary; Edward L. Holmes, Treasurer.

*Trustees.*—Walter L. Newberry, William H. Brown, Charles V. Dyer, Luther Haven, Ezra B. McCagg, William Barry, Flavel Moseley, Samuel Stone, Philo Carpenter, Rev. N. L. Rice, D.D., John H. Kinzie and Mark Skinner.

*Board of Surgeons.—Trustees ex-Officio.*—Consulting Surgeons, Prof. Daniel Brainard, M.D., Prof. Joseph W. Freer, M.D.; Attending Surgeons, Edward L. Holmes, M.D., Wm. H. Baltzell, M.D.

